# Supplementary material for: Identification of photoperiod-induced specific miRNAs in the adrenal glands of Sunite sheep (Ovis aries)
Source: Front Vet Sci. 2022 Jul 22;9:888207. doi: 10.3389/fvets.2022.888207 (PMC9354845; doi:10.3389/fvets.2022.888207)
Supplement: Supplementary file 1 [file Data_Sheet_1.ZIP › Supplementary file/miRDeep2 file.zip!/miRDeep2 file/Know_Structure/SP42A3/oar-mir-29a.pdf]

miRBase precursor : oar-mir-29a
Total read count : 122102
oar-miR-29a read count : 122080
remaining reads : 22

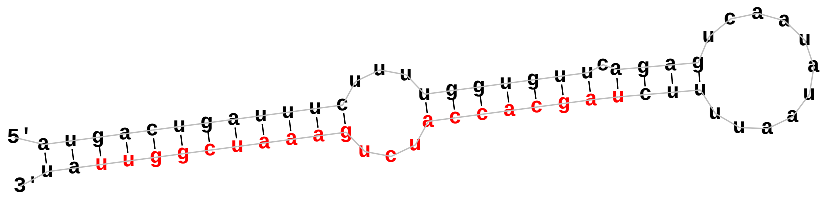

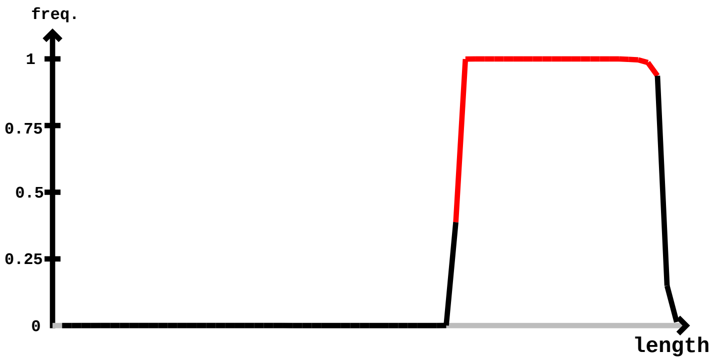


```
                                                oar-miR-29a
5'- augacugauuucuuuugguguucagagucaauauaauuuucuagcaccaucugaaaucgguuau   -3'    exp
    ((((((((((((...(((((((.((((...........)))))))))))...))))))))))))    reads      mm         sample
    .ugacugauuucuuuugguguuc.........................................    7          0          seq
    ...acugauuucuuuugguguuc.........................................    13         0          seq
    ..........................gucaauauaauuuucuagcaccaucugaaaucgguua.    1          0          seq
    ...........................ucaauauaauuuucuagcaccaucugaaaucgguu..    1          0          seq
    .......................................ucuagcaccaucugaaaucg.....    1          0          seq
    .......................................ucuagcaccaucugaaaucgg....    5          0          seq
    .......................................ucuagcaccaucugaaaucggu...    18         0          seq
    .......................................ucuagcaccauUugaaaucgguu..    1          1          seq
    .......................................ucuagcaccaucugaaaucgguu..    12         0          seq
    .......................................ucuagcaccaucugaaaucgguua.    1          0          seq
    ........................................cuagcaccaucugaaau.......    5          0          seq
    ........................................cuagcaccaucugaaaA.......    1          1          seq
    ........................................cuagcaccaucugaaauA......    1          1          seq
    ........................................cuagcaccaucugaaauc......    32         0          seq
    ........................................cuagcaccaucugaaaucg.....    43         0          seq
    ........................................cuagcaccauUugaaaucgg....    2          1          seq
    ........................................cuagcaccaucugaaaucgg....    189        0          seq
    ........................................cuagcaccauUugaaaucggu...    21         1          seq
    ........................................cuagcaccaucugaaaucggu...    1689       0          seq
    ........................................cuagcaccaucugaaaucgguu..    43553      0          seq
    ........................................cuagcaccauUugaaaucgguu..    192        1          seq
    ........................................cuagcaccauUugaaaucgguua.    4          1          seq
    ........................................cuagcaccaucugaaaucgguua.    1440       0          seq
    ........................................cuagcaccaucugaaaucgguuau    137        0          seq
    ........................................cuagcaccauUugaaaucgguuau    1          1          seq
    .........................................uagcaccaucugaaauU......    10         1          seq
    .........................................uagcaccaucugaaauA......    10         1          seq
    .........................................uaUcaccaucugaaauc......    1          1          seq
    .........................................uagcaccaucugaaGuc......    1          1          seq
    .........................................uagcaccaucugaaauc......    139        0          seq
    .........................................uagcaccaucugaaaucg.....    161        0          seq
    .........................................uagcaccauUugaaaucg.....    22         1          seq
    .........................................uagcaccaucugaaaucgg....    960        0          seq
    .........................................uagcaccauUugaaaucgg....    71         1          seq
    .........................................uagcaccauUugaaaucggu...    515        1          seq
```

oar-miR-29a

```
augacugauuucuuuugguguucagagucaauauaauuuucuagcaccaucugaaaucgguuau
.........................................uagcaccaucugaaaucggu...   3812    0    seq
.........................................uagcaccauUugaaaucgguu..   2505    1    seq
.........................................uagcaccaucugaaaucgguu..   49845   0    seq
.........................................uagcaccauUugaaaucgguua.   776     1    seq
.........................................uagcaccaucugaaaucgguua.   14356   0    seq
.........................................uagcaccauUugaaaucgguuau   71      1    seq
.........................................uagcaccaucugaaaucgguuau   1413    0    seq
..........................................agcaccaucugaaaucgg....   1       0    seq
..........................................agcaccaucugaaaucggu...   2       0    seq
..........................................agcaccaucugaaaucgguu..   45      0    seq
..........................................agcaccauUugaaaucgguu..   1       1    seq
..........................................agcaccaucugaaaucgguua.   10      0    seq
..........................................agcaccaucugaaaucgguuau   1       0    seq
...........................................gcaccaucugaaaucgguu..   3       0    seq
...........................................gcaccaucugaaaucgguua.   1       0    seq
```
